# Children’s Health: Do Antibiotics Now Mean Asthma Later?

**Published:** 2006-06

**Authors:** Carol Potera

Asthma affects 1 in 8 school-aged children in industrialized countries, making it the most common chronic illness in this group. Now a meta-analysis of child asthma studies led by pharmaceutical scientist Fawziah Marra of the University of British Columbia shows that children diagnosed with asthma were twice as likely as nonasthmatics to have received antibiotics before age 1. The more courses of antibiotics a child received in the first year of life, the higher the risk for asthma.

The meta-analysis, reported in the March 2006 issue of *Chest*, examined the link between antibiotic exposure in babies and subsequent development of asthma, as well as the dose–response relationship. Marra’s team analyzed four prospective studies and four retrospective studies conducted between 1999 and 2004. Each study involved between 263 and 21,120 children, including cases who had been diagnosed with asthma between the ages of 1 and 18 years. The number of antibiotic courses taken ranged from one to seven, and averaged three.

Pooling the data from all eight studies revealed a twofold risk of developing asthma with at least one course of antibiotics. Each additional course raised asthma risk 1.16 times. Information about the antibiotics prescribed could not be obtained from the studies.

The findings support the “hygiene hypothesis,” which proposes that an immune system that doesn’t get enough practice killing germs (due to either an excessively clean environment or overuse of antibiotics) will become overly sensitized and overreact to normally harmless environmental agents such as pollen and dust.

Marra and her colleagues recently launched a community education campaign in British Columbia called “Do Bugs Need Drugs?” The program uses media ads, classroom visits, and educational materials to teach health professionals and the general public about the overuse of antibiotics. The campaign emphasizes the difference between bacterial and viral infections, useful preventive measures such as hand washing, and the need to use antibiotics wisely. “In children, antibiotics are commonly used to treat ear infections, upper respiratory tract infections, and bronchitis,” says Marra, even though many such infections are viral and don’t respond to antibiotics. Some parents may refuse to leave a doctor’s office without a prescription.

The information gained from the meta-analysis is valuable for physicians who are striving to cut back on prescribing antibiotics, says W. Michael Alberts, president of the American College of Chest Physicians: “It can help to convince parents of young children to hold off on giving antibiotics unless absolutely necessary.”

## Figures and Tables

**Figure:**
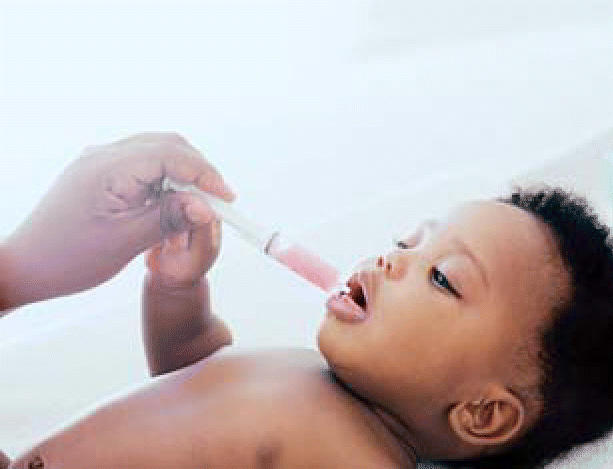
Unpleasant side effect Antibiotic use before age 1 could contribute to childhood asthma.

